# Evidence of gene nucleotide composition favoring replication and growth in a fastidious plant pathogen

**DOI:** 10.1093/g3journal/jkab076

**Published:** 2021-03-14

**Authors:** Andreina I Castillo, Rodrigo P P Almeida

**Affiliations:** Department of Environmental Science, Policy and Management, University of California, Berkeley, CA 94720, USA

**Keywords:** *Xylella fastidiosa*, nucleotide composition, GC content, information storage and processing (ISP)

## Abstract

Nucleotide composition (GC content) varies across bacteria species, genome regions, and specific genes. In *Xylella fastidiosa*, a vector-borne fastidious plant pathogen infecting multiple crops, GC content ranges between ∼51-52%; however, these values were gathered using limited genomic data. We evaluated GC content variations across *X. fastidiosa* subspecies *fastidiosa* (*N* = 194), subsp. *pauca* (*N* = 107), and subsp. *multiplex* (*N* = 39). Genomes were classified based on plant host and geographic origin; individual genes within each genome were classified based on gene function, strand, length, ortholog group, core *vs* accessory, and recombinant *vs* non-recombinant. GC content was calculated for each gene within each evaluated genome. The effects of genome and gene-level variables were evaluated with a mixed effect ANOVA, and the marginal-GC content was calculated for each gene. Also, the correlation between gene-specific GC content *vs* natural selection (dN/dS) and recombination/mutation (r/m) was estimated. Our analyses show that intra-genomic changes in nucleotide composition in *X. fastidiosa* are small and influenced by multiple variables. Higher AT-richness is observed in genes involved in replication and translation, and genes in the leading strand. In addition, we observed a negative correlation between high-AT and dN/dS in subsp. *pauca*. The relationship between recombination and GC content varied between core and accessory genes. We hypothesize that distinct evolutionary forces and energetic constraints both drive and limit these small variations in nucleotide composition.

## Introduction

The relevance of nucleotide composition (GC content) in genome evolution has been well established from a genomics (Arndt *et al.* 2005; Amit *et al.* 2012; Šmarda *et al.* 2014; Mugal *et al.* 2015; Almpanis *et al.* 2018), ecological (Bolhuis *et al.* 2006; Šmarda *et al.* 2014; Luo *et al.* 2015), and biological perspective (Mann and Chen 2010; Udaondo *et al.* 2016; Bohlin *et al.* 2017; Du *et al.* 2018; Castillo *et al.* 2019a). Specifically in proteobacteria, whole-genome GC content varies between 17% and 75% (Brocchieri 2014), and multiple evolutionary mechanisms have been proposed to explain this variation. One mechanism is GC-biased gene conversion (gBGC), which refers to a repair bias favoring GC over AT alleles during recombination (Galtier *et al.* 2001). This mechanism describes how highly recombinant genome regions, or organisms where recombination is more frequent, tend to be more GC-rich (Lassalle *et al.* 2015; Romiguier and Roux 2017). Specific nucleotide compositions can also be favored by natural selection. For example, the energetic cost of synthesizing A/T nucleotides is considered lower than that of synthesizing G/C nucleotides (Du *et al.* 2018). Hence, nucleotide composition differences in energetically constrained environments are explained by the energetic limitations to nucleotide synthesis (Li *et al.* 2015). Genetic GC content is linked to gene expression; in bacteria higher GC content is correlated with higher expression (Zhou *et al.* 2014; Chen *et al.* 2016) and fitness (Raghavan *et al.* 2012). Intra-genic changes in GC content have also been associated with mRNA stability; GC content is reduced near the start codon of protein-coding genes, particularly in those with higher average GC content, as a mechanism to facilitate protein translation (Gu *et al.* 2010). Finally, an important factor affecting GC-content is mutation bias. With some exceptions (McCutcheon *et al.* 2009; Dillon *et al.* 2015), mutation in bacterial genomes is AT-biased (Hershberg and Petrov 2010). It is believed that this is a result of a bias toward transitions at fourfold degenerate sites (Hildebrand *et al.* 2010) with C to T and G to A transitions being consistently favored over T to C and A to G transitions, even in GC-rich genomes (Hershberg and Petrov 2010). The bias is particularly evident in obligate symbionts due to loss of DNA repair genes (Moran *et al.* 2008) and small effective population size (Balbi *et al.* 2009). Moreover, balances between mutation trends and natural selection have a strong impact on nucleotide composition. For example, the GC content of substituted bases (sbGC) has been found to be GC biased compared with the GC content of the core genome in most microbial genomes (except for those highly GC-rich). This trend has been proposed to be linked to natural selection countering the C/G to T/A transitional bias universally observed in microbial genomes (Bohlin *et al.* 2018).

Overall, no single mechanism is likely to fully explain changes in nucleotide composition. Instead, GC content variations likely stem from interactions and balances among adaptive and non-adaptive forces (*e.g.* mutation *vs* selection). As a result, distinct genes, gene/genome regions, genomes, and populations can achieve unique GC content values (Botzman and Margalit 2011). Studying changes in GC content can highlight general trends, pinpoint taxa not following those trends, and illustrate evolutionary mechanisms relevant for a group. In this regard, while it is known that bacterial genome composition follows unique evolutionary trends; most analyses have been largely skewed toward species of clinical and veterinary interest (Lassalle *et al.* 2015; Bohlin *et al.* 2017). In terms of plant-associated bacteria, only 0.1% of the estimated 3 million taxa have been studied (Ingram 2002), with pathogens affecting the agricultural and forestry industries being overrepresented (Mansfield *et al.* 2012; Almeida 2018). These studies are commonly aimed to describe aspects of biogeography, host-pathogen adaptive potential, or pathogen management. Because of this, there is little to no research characterizing changes in plant-pathogen genome structure and nucleotide composition, despite its undisputed evolutionary role.

Both intra- and inter-genomic GC content variations have been identified in specific bacterial plant symbionts. Changes in intra-genomic GC content are indicative of a highly flexible genome in *Xanthomonas campestris* (Thieme *et al.* 2005). Also, similarities between genomic GC content within *Erwinia* and *Enterobacter* suggest that, contrary to expectation, nucleotide composition is preserved across the free living and obligate symbiont lifestyles (Estes *et al.* 2018). These are indicators that GC content in bacterial plant-associated bacteria has unique intra- and inter-genomic trends worthy of exploring. However, there are too few studies focused on the matter to highlight any pattern. Creating a comprehensive analysis in GC content variation is unfeasible, but this knowledge gap could be breached by characterizing pivotal groups. In this regard, *Xylella fastidiosa*, an emerging vector-borne plant pathogen with an expanding host and geographic range (EFSA 2018), represents a key study system due to its obligate colonization of plant and insect vector hosts.

Compared with its closest relative (*i.e. Xanthomonas* spp.), *X. fastidiosa* has undergone substantial genomic and biological changes. The *X. fastidiosa* genome is half the size of *Xanthomonas* spp. and characterized by the loss of specific metabolic functions and a slow growth rate (Gerlin *et al.* 2020). Unlike *Xanthomonas* spp., *X. fastidiosa* is transmitted by xylem sap-feeding insects (Vicente and Holub 2013; Cornara *et al.* 2017; Overall and Rebek 2017). Multiple studies have highlighted how changes in lifestyle influence genomic nucleotide composition (Foerstner *et al.* 2005; Merhej *et al.* 2009; Mann and Chen 2010; Dutta and Paul 2012; Aslam *et al.* 2019). In particular, lower GC content can result from drops in effective population size (Lassalle *et al.* 2015) and nutritional limitations (Mann and Chen 2010), both of these are variables found in vector-borne pathogens. In addition, *X. fastidiosa* has been introduced to multiple naïve crop populations (Schuenzel *et al.* 2005; Nunney *et al.* 2013; Landa *et al.* 2020) where it is hypothesized to evolve clonally (Ramazzotti *et al.* 2018; Sicard *et al.* 2018). This geographic component could affect nucleotide composition, since previous studies have highlighted how stronger AT-biases are observed in clonal bacterial populations due to the relaxation natural selection (Hershberg and Petrov 2010). For these reasons, characterizing GC content in *X. fastidiosa* might aid in better understanding of how evolutionary and biological forces shape the genomes of plant-associated bacteria.

The available *X. fastidiosa* genomes reported an average GC content of 51–52% (Simpson 2000; Castillo *et al.* 2019b); however, no study has evaluated inter-genic variations in GC content, defined the evolutionary forces shaping nucleotide composition, or compared these trends with those of other phytopathogens. We examined gene-specific GC content variations in subsp. *pauca*, subsp. *multiplex*, and subsp. *fastidiosa*. We evaluated if changes in GC content were associated with ecological (*i.e.* host plant, geographic source, etc.), functional (*i.e.* clusters of orthologous group [COGs]), evolutionary (*i.e.* selection, core/accessory, or recombinant/non-recombinant genes), or genetic variables (*i.e.* gene position, gene length, gene strand, etc.). Finally, we compared the GC content of *X. fastidiosa* with four other plant pathogens: *Xanthomonas citri*, *X. campestris*, *Xanthomonas oryzae* pv*. oryzae*, and *Xanthomonas oryzae* pv*. oryzicola* (which are closely related to *X. fastidiosa*), and the more distant *Agrobacterium tumefaciens*.

## Materials and methods

### Whole-genome sequences from worldwide *X. fastidiosa* isolates, and publicly available *X. citri*, *X. camprestri*, *X. oryzae pv. oryzae*, *X. oryzae pv. oryzicola*, and *A. tumefaciens* isolates

The following study encompasses 340 *X. fastidiosa* whole-genome sequences. Isolates were collected from infected plant material in diverse geographic regions. Isolates belong to subsp. *fastidiosa* (*N* = 194), subsp. *multiplex* (*N* = 39), and subsp. *pauca* (*N* = 107). The number of isolates varied among geographic locations and infected plant hosts. Raw data have been made publicly available (Supplementary Table S1). Most isolates were sequenced using Illumina HiSeq2000; however, five subsp. *fastidiosa* isolates were sequences using both Illumina HiSeq2000 and PacBio (Castillo *et al.* 2020); and five subsp. *pauca* isolates were sequenced from total plant DNA (Sicard *et al.* in preparation). Samples were sequenced at the University of California, Berkeley Vincent J. Coates Genomics Sequencing Laboratory (California Institute for Quantitative Biosciences; QB3). The quality of the genome assemblies varied but coverage remained above ∼59×.

Details on genome assembly and annotation protocols have been provided in previous studies (Castillo *et al.* 2019b, 2020; Landa *et al.* 2020). Briefly, the quality of raw paired FASTQ reads was evaluated using FastQC (Andrews and Wingett 2018) and visualized using MultiQC (Ewels *et al.* 2016). Following, Seqtk v1.2 (https://github.com/lh3/seqtk) and cutadapt v1.14 (Marcel 2011) were used to remove low-quality reads and adapter sequences, respectively. Pre-processed reads were assembled *de novo* with SPAdes v3.13 (Bankevich *et al.* 2012; Nurk *et al.* 2013). Assembled contigs were reordered with Mauve’s contig mover function (Rissman *et al.* 2009) using complete publicly available assemblies as references (Temecula1 (GCA_000007245.1) for subsp. *fastidiosa*, 9a5c (ASM672v1) for subsp. *pauca*, and M12 (ASM1932v1) for subsp. *multiplex*). Finally, genomes were annotated using Prokka (Seemann 2014). Contamination was suspected on subsp. *fastidiosa* isolate XF70 from Costa Rica and removed by mapping FASTQ reads to its closest phylogenetic relative as described by Castillo *et al.* (2020). In the case of isolates obtained from total plant DNA, QC FASTQ reads were mapped to the *Olea europaea* genome assembly (GCA_900603015.1) with bowtie2 v2.3.4.1 (Langmead and Salzberg 2012). A SAM file of paired unmapped reads was created using the −f 12 and −F 256 flags in Samtools v1.8 (Li *et al.* 2009) and subsequently converted to sorted BAM. Bedtools v2.26.0 (Quinlan and Hall 2010) was used to convert the sorted BAM file into FASTQ files. The host-removed reads were then assembled with SPAdes v3.13 as described previously. In addition, publicly available whole-genome assemblies for *A. tumefaciens* (*N* = 12), *X. oryzae pv. oryzae* (*N* = 75), *X. oryzae pv. oryzicola* (*N* = 13), *X. campestris* (*N* = 14), and *X. citri* (*N* = 68) were obtained from NCBI and re-annotated with Prokka. Metadata for these isolates have also been included in Supplementary Table S1.

### Pan-genome analysis of phytopathogen groups

Roary v3.11.2 (Page *et al.* 2015) was used to calculate the size of the core (genes shared between 99 and 100% strains), soft-core (genes shared between 95 and 99% strains), shell (genes shared between 15 and 95% strains), and cloud (genes shared between <15% strains) genomes for each *X. fastidiosa* subspecies, *A. tumefaciens*, *X. oryzae* pv*. oryzae*, *X. oryzae* pv*. oryzicola*, *X. campestris*, and *X. citri*. The soft-core, shell, and cloud genomes were compiled into the accessory genome (genes shared between <99% strains). Individual genome assemblies and annotation files were used to mine gene sequences within each genome. Individual genes were also classified regarding their number of orthologs. Finally, all individual genes were categorized based on their COGs (Tatusov 2000). Each gene was classified by its specific COG function (*e.g.* translation and ribosomal structure and biogenesis, transcription, nuclear structure, cell motility, lipid transport, and metabolism, etc.) and by the main categories to which these functions belong [*i.e.* Metabolism (M), Information Storage and Processing (ISP), and Cellular Processes and Signaling (CPS)]. Individual genes without defined COG (*e.g.* hypothetical proteins) were assigned to the Poorly characterized (P) category. Individual genes belonging to two or more functional classes were grouped as Multiple categories (MU). Approximately 30–40% of individual genes were classified within the P category.

### Recombination detection and estimation of global genetic diversity in core alignments

Roary was used to create core genome alignments for each *X. fastidiosa* subspecies, *A. tumefaciens*, *X. oryzae* pv*. oryzae*, *X. oryzae* pv*. oryzicola*, *X. campestris*, and *X. citri*. The global measure of genetic diversity (π) was calculated for the core genome alignment of each phytopathogen using the R package “PopGenome” (Pfeifer *et al.* 2014). Nucleotide diversity (π) measures the average number of nucleotide differences per site in pairwise comparisons among DNA sequences. This measurement has been used to characterize *X. fastidiosa* populations (Vanhove *et al.* 2019, 2020; Castillo *et al.* 2020), and thus, can be easily contrasted to earlier studies.

FastGEAR (Mostowy *et al.* 2017) was used with default parameters to identify lineage-specific (ancestral) and strain-specific (recent) recombinant segments in *X. fastidiosa* subspecies core genome alignments. A custom python script was used to find individual core genes contained entirely within recombinant segments. Core genes found entirely within recombinant regions were henceforth classified as “recombinant genes,” and the remaining genes were classified as “non-recombinant genes.” The correlation between GC content and the recombination/mutation rate (r/m) was also evaluated for individual gene alignments. Briefly, Roary was used to identify individual ortholog groups within each *X. fastidiosa* subspecies. The detected ortholog groups were programmatically aligned using MACSE v2 (Ranwez *et al.* 2018). The corresponding Maximum Likelihood (ML) trees were then built with RAxML (Stamatakis 2014). All trees were built using the GTRCAT substitution model and tree topology and branch support were assessed with 100 bootstrap replicates. Each gene alignment and tree were then used as input for ClonalFrameML (Didelot and Wilson 2015). A subspecies-wide Pearson correlation coefficient was performed between GC content and the log10 transformation of r/m.

### Estimation of gene-specific GC content and statistical analysis

A custom python script was used to calculate gene-specific GC content values for each gene within individual *X. fastidiosa*, *A. tumefaciens*, *X. oryzae* pv*. oryzae*, *X. oryzae* pv*. oryzicola*, *X. campestris*, and *X. citri* genomes. GC content in the third or wobble position (GC_3_) was also calculated for each gene within individual *X. fastidiosa* subspecies. The length of each gene was calculated based on its start and end positions. Variables were assigned as gene functional class, gene size, gene strand, accessory/core gene, number of orthologs, and recombinant/non-recombinant gene (gene-dependent); and geographic location and plant host (genome-dependent). Certain variables known to affect genomic GC content were not studied here. For example, large scope RNAseq data were not available at the time of analysis, precluding any assessment of gene expression levels and their relationship to GC content. It is expected that this type of data will become available in the future. Similarly, most of the whole genome sequences used have been assembled to the contig level, and thus, it was not possible to estimate gene position in a full chromosome. In this regard, gene position was obtained for three finished representative whole-genome assemblies: Temecula1 (GCA_000007245.1) for subsp. *fastidiosa*, 9a5c (ASM672v1) for subsp. *pauca*, and M12 (ASM1932v1) for subsp. *multiplex*. A synteny analysis among the assemblies was conducted using CoGe (https://genomevolution.org/coge/ [last accessed on March 19th, 2021]).

A mixed-effect ANOVA was used to evaluate the statistical contributions of gene-dependent and genome-dependent variables in the nucleotide composition of individual genes. The Genome ID and genome GC content were used as a random slope and the gene ortholog group identified by Roary was used as a random effect. The model was used to estimate marginal means for gene-specific GC content (marginal-GC). In other words, the marginal-GC content representing the estimated GC content values averaged across all variable levels of the linear regression model.

### Correlation between genic GC content with dN/dS values

Orthologous genes were identified, aligned, and their ML trees were constructed as described in the “Recombination detection and estimation of global genetic diversity in core alignments” section of the Materials and methods. The relationship between GC content and gene-wide signs of selection was estimated. The rate of non-synonymous over synonymous substitutions (dN/dS) was programmatically calculated for each gene alignment using BUSTED (Kosakovsky Pond *et al.* 2005; Murrell *et al.* 2015). Briefly, BUSTED determines if there is at least one positively selected site across the alignment and in any branch of the phylogeny. The subspecies-wide Pearson’s correlation coefficient was calculated between GC content and the Tukey’s Ladder of Powers transformation of dN/dS values.

### Data availability

Raw sequence files are available upon request. Published sequence data are available at GenBank; the accession numbers are listed in Supplementary Table S1. Supplementary material is available at figshare: https://doi.org/10.25387/g3.14067449.

## Results

### GC content variations are more readily observed within the accessory genome

**Figure 1:**
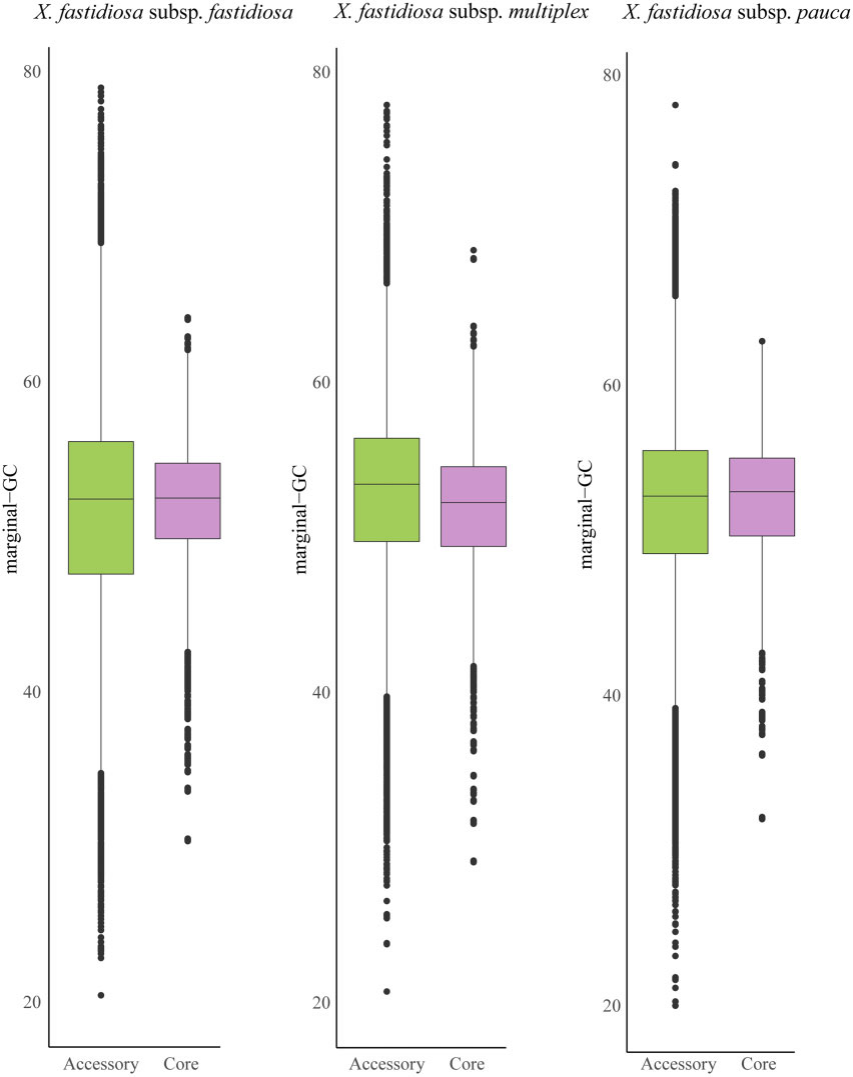
Boxplot showing differences in marginal-GC content in core *vs* accessory genes in three *X. fastidiosa* subspecies.

Core and accessory genome sizes varied within *X. fastidiosa* subspecies and in the other phytopathogens examined (Table 1). The standard deviation of genic GC content was lower in core genes compared with accessory genes (Figure 1 and Table 2). Gene-specific GC content also varied within accessory genome components for all three subspecies (Table 2). Specifically, genes in the shell genome had higher GC content variation compared with soft-core and cloud genomes in subsp. *fastidiosa* and subsp. *pauca* (Supplementary Figure S1). It should be noted that the shell genome has the widest range in relation to the percentage of strains sharing a given gene (15–95%). Therefore, it can potentially cover a wider range of gene presence/absence variations compared with the soft-core (genes shared by 95–99% of strains) and the cloud genomes (genes shared by <15% of strains).

**Table 1:** Number of genes within each pan-genome component

Phytopathogen	*N*	Genome component			
		Core	Soft-core	Shell	Cloud
*Xylella fastidiosa* subsp. *fastidiosa*	194	1,488	297	849	7,644
*Xylella fastidiosa* subsp. *multiplex*	39	1,006	226	2,152	5,982
*Xylella fastidiosa* subsp. *pauca*	107	1,244	470	1,100	6,513
*Xylella oryzae* pv*. oryzae*	75	2,460	311	3,812	4,824
*Xylella oryzae pv. oryzicola*	13	3,391	0	2,277	1,316
*Xylella citri*	68	1,084	1,887	2,444	11,027
*Xylella campestris*	14	3,372	0	1,664	1,987
*Agrobacterium tumefaciens*	12	1,638	0	9,774	11,009

*N* = number of genomes.

**Table 2:** (a) Mean GC content and standard deviation (sd) for genes in the core, accessory, and accessory genome components (soft-core, shell, and cloud genome) across subspecies. (b) F-test shows statistical significance of variance between groups

Subspecies	Core	Accessory	Accessory components		
			Soft-core	Shell	Cloud
*Xylella fastidiosa* subsp. *fastidiosa*	51.90 (4.37)	51.56 (7.28)	51.87 (6.01)	51.31 (8.19)	52.83 (9.16)
*Xylella fastidiosa* subsp. *multiplex*	51.52 (4.78)	52.49 (6.33)	52.23 (5.36)	52.47 (6.38)	52.93 (7.39)
*Xylella fastidiosa* subsp. *pauca*	52.51 (4.56)	52.19 (6.17)	52.23 (4.81)	52.05 (7.73)	52.32 (8.24)
Subspecies	Core *vs* accessory		Soft-core vs shell	Shell vs cloud	Cloud vs Soft-core
*Xylella fastidiosa* subsp. *fastidiosa*	*F* = 0.360		*F = 0.539*	*F = 1.251*	*F = 2.320*
	*P* < 2.2 × 10^−16*^		P < 2.2 × 10−16*	*P* < 2.2 × 10^−16*^	P < 2.2 × 10−16*
*Xylella fastidiosa* subsp. *multiplex*	*F* = 0.571		*F = 0.706*	*F = 1.343*	*F = 1.903*
	*P* < 2.2 × 10^−16*^		P < 2.2 × 10−16*	*P* < 2.2 × 10^−16*^	P < 2.2 × 10−16*
*Xylella fastidiosa* subsp. *pauca*	*F* = 0.546		*F = 0.386*	*F = 1.135*	*F = 2.935*
	*P* < 2.2 × 10^−16*^		P < 2.2 × 10−16*	*P* < 2.2 × 10^−16*^	P < 2.2 × 10−16*

Bold values correspond to p-values < 0.05. These values have also been marked with asterisk (*). *Statistically significant differences.

The nucleotide composition in cloud, shell, and soft-core genes was similar; however, there was a small and statistically significant decrease in GC content in soft-core genes relative to cloud genes (*t* = 14.866, *P* < 2.2 **×** 10^−16^ for subsp. *fastidiosa*; and *t* = 7.1555, *P* = 8.774 **×** 10^−13^ for subsp. *multiplex*) and in shell genes relative to cloud genes (*t* = 22.975, *P* < 2.2 **×** 10^−16^ for subsp. *fastidiosa*; *t* = 4.9127, *P* = 9.111 **×** 10^−07^ for subsp. *multiplex*; and *t* = 4.2695, *P* = 1.963 **×** 10^−05^ for subsp. *pauca*). However, this was not seen between the soft-core genes relative to cloud genes in subsp. *pauca* (*t* = 1.6353, *P* = 0.102). Trends in GC content variation based on explanatory variables are discussed below.

Apart from the variable “Host plant” and “Population,” most gene- and genome-dependent variables affected GC content (Table 3). This pattern was observed when using accessory and core gene classifications made by different pan-genome analysis programs [Roary *vs* GET_HOMOLOGUES (Contreras-Moreira and Vinuesa 2013)], when Hypothetical/Poorly characterized genes were removed from the dataset, and when Roary’s core genome threshold was reduced to 95% (Supplementary Table S2). GC_3_ variation followed the same trends (Supplementary Table S3). Statistically significant gene- and genome-dependent variables were subsequently plotted to evaluate if biological or ecological trends were present. In addition, the size effect (Cohen’s *d*) was calculated for the three statistically significant categorical variables: Accessory *vs* Core, DNA strand, and gene functional class (Table 4).

**Table 3:** Mixed effect ANOVA results on gene-specific GC content

Subspecies	GC									
	Genome				Accessory			Core		
*Xylella fastidiosa* subsp. *fastidiosa*	Variable	Chisq	Df	Pr(>Chisq)	Chisq	Df	Pr(>Chisq)	Chisq	Df	Pr(>Chisq)
	Accessory/Core	171.3993	1	<2 × 10^−16*^	–	–	–	–	–	–
	Host	162.6594	8	<2 × 10^−16*^	75.9588	8	3.17 × 10^−13*^	80.8329	8	3.32 × 10^−14*^
	Gene length	11749.963	1	<2 × 10^−16*^	7514.8866	1	<2 × 10^−16*^	417.0152	1	<2 × 10^−16*^
	Strand	914.5679	1	<2 × 10^−16*^	420.8673	1	<2 × 10^−16*^	934.1671	1	<2 × 10^−16*^
	Population^a^	7.4435	7	0.3842	5.1146	7	0.646	0.7336	7	0.9981
	Function	1,801.8669	4	<2 × 10^−16*^	631.8232	4	<2 × 10^−16*^	10,113.0656	4	<2 × 10^−16*^
	Number of orthologs^b^	6,871.6529	189	<2 × 10^−16*^	2,462.0721	189	<2 × 10^−16*^	–	–	–
*Xylella fastidiosa * subsp. *multiplex*	Accessory/Core	14.5632	1	**0.0001** ^*^	–	–	–	–	–	–
	Host	14.0509	15	0.5217	12.4927	15	0.6414	14.0804	15	0.5194
	Gene length	434.2259	1	<2 × 10^−16*^	255.1272	1	<2 × 10^−16*^	97.9301	1	<2 × 10^−16*^
	Strand	60.102	1	9.01 × 10^−15*^	20.179	1	7.05 × 10^−06*^	134.2912	1	<2 × 10^−16*^
	Population^a^	5.5217	5	0.3556	6.0869	5	0.2979	1.3441	5	0.9303
	Function	265.9274	4	<2 × 10^−16*^	196.8562	4	<2 × 10^−16*^	81.2937	4	<2 × 10^−16*^
	Number of orthologs^b^	597.4762	37	<2 × 10^−16*^	354.634	37	<2 × 10^−16*^	–	–	–
*Xylella fastidiosa * subsp. *pauca*	Accessory/Core	60.515	1	7.30 × 10^−15*^	–	–	–	–	–	–
	Host	5.153	5	0.3975	0.7541	5	0.9799	1.0277	5	0.9603
	Gene length	3,677.413	1	<2 × 10^−16*^	3,060.2776	1	<2 × 10^−16*^	758.9033	1	<2 × 10^−16*^
	Strand	567.02	1	<2 × 10^−16*^	457.0479	1	<2 × 10^−16*^	76.3242	1	<2 × 10^−16*^
	Population^a^	2.298	3	0.5129	1.2317	3	0.7454	3.3509	3	0.3406
	Function	253.732	4	<2 × 10^−16*^	326.8554	4	<2 × 10^−16*^	118.7646	4	<2 × 10^−16*^
	Number of orthologs^b^	3,105.453	103	<2 × 10^−16*^	1,954.636	103	<2 × 10^−16*^	–	–	–

Bold values correspond to p-values < 0.05. These values have also been marked with asterisk (*). *Statistically significant differences.

a Refers to the geographic populations included in this study: California, Southeastern USA, Taiwan, Spain, Brazil, Italy, Costa Rica, and France.

b Refers to the number of orthologues for each gene.

**Table 4:** Cohen’s *d* of the accessory/core, DNA strand, and COG function classes

Subspecies	Variables		Cohen’s *d*	95% CI
*Xylella fastidiosa* subsp. *fastidiosa*	Accessory/Core		−0.0571	CI: (−0.063, −0.0513)
	Strand		0.0392	CI: (0.0333, 0.045)
	Function	M *vs* CPS	−0.3202	CI: (−0.3328, −0.3078)
		M *vs* ISP	−0.5112	CI: (−0.5236, −0.4988)
		ISP *vs* CPS	0.2308	CI: (0.2168, 0.2450)
*Xylella fastidiosa* subsp. *multiplex*	Accessory/Core		0.167	CI: (0.1526, 0.1814)
	Strand		0.0504	CI: (0.0364, 0.0645)
	Function	M *vs* CPS	−0.345	CI: (−0.3757, −0.3144)
		M *vs* ISP	−0.4868	CI: (−0.5169, −0.4567)
		ISP *vs* CPS	0.1822	CI: (0.1477, 0.2167)
*Xylella fastidiosa* subsp. *pauca*	Accessory/Core		−0.0553	CI: (−0.0649, −0.0456)
	Strand		0.0238	CI: (0.0160, 0.0317)
	Function	M *vs* CPS	−0.371	CI: (−0.3879, −0.3542)
		M *vs* ISP	−0.5116	CI: (−0.5284, −0.4949)
		ISP *vs* CPS	0.1874	CI: (0.1684, 0.2064)

### Nucleotide composition varies between the leading and lagging DNA strand

The average number of individual genes in the lagging and leading strands was 1055 *vs* 1030 for subsp. *fastidiosa*, 1096 *vs* 1095 for subsp. *pauca*, and 1127 *vs* 1079 for subsp. *multiplex*. Overall, genes in the leading strand had lower marginal-GC content than those in the lagging strand in the core genome of subsp. *multiplex* and *pauca*, and the accessory genome of subsp. *fastidiosa* (Supplementary Figure S2). In addition to marginal-GC, GT was calculated for individual genes in the leading and lagging strand in all *X. fastidiosa* subspecies (Supplementary Figure S3). The goal was to establish if G and T nucleotides were enriched in either DNA strand (Lobry and Sueoka 2002). GT content was higher in the lagging strand of subsp. *fastidiosa* core genome and the core and accessory genomes of subsp. *pauca*.

### ISP genes show lower GC content distribution

**Figure 2:**
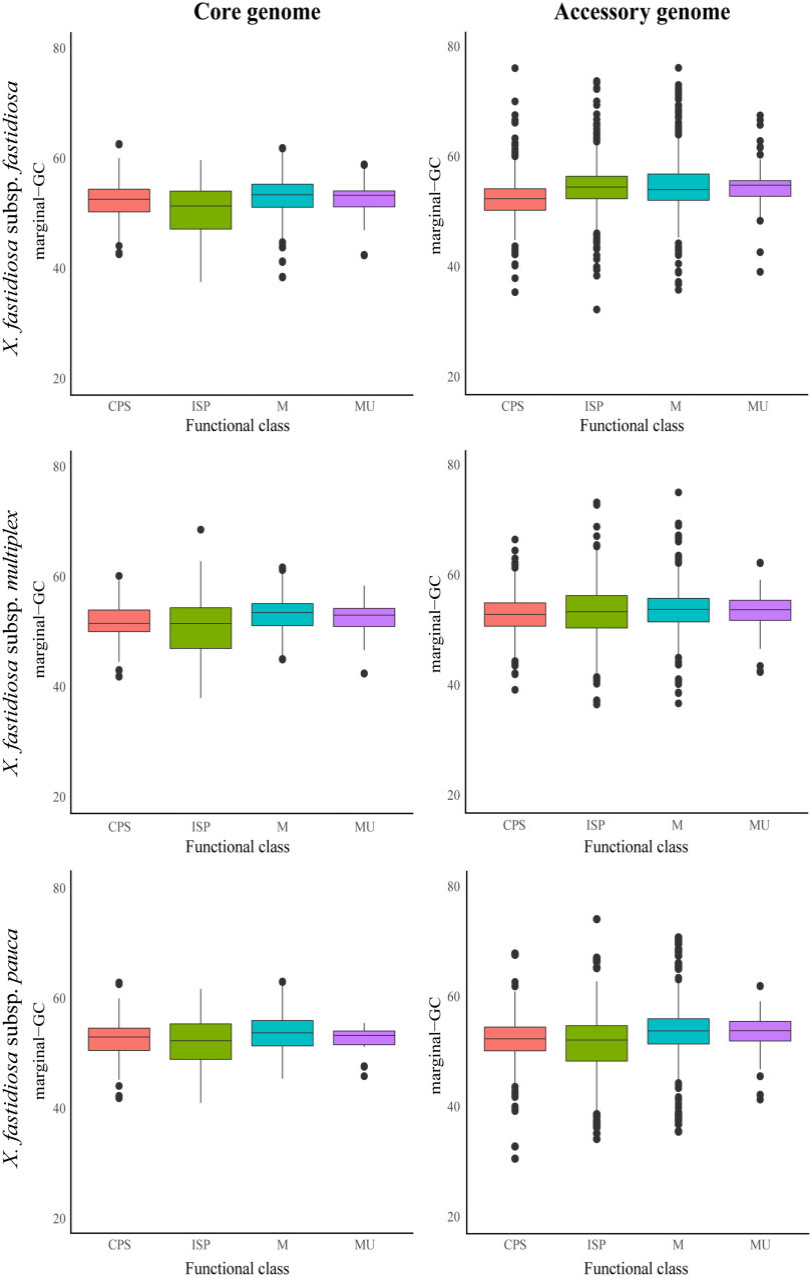
Boxplot showing marginal-GC content distribution on different functional classes for three *X. fastidiosa* subspecies. Plots have been divided into core/accessory genes within each *X. fastidiosa* subspecies. Functional classes include ISP (green), Cellular Processes and Signaling (CPS, blue), Metabolism (M, red), and Multiple categories (MU, purple).

Gene functional class also affected genic GC content. The number of core and accessory genes from different functional groups varied within subspecies. The Metabolism (M) functional class was the most numerous, while the CPS and ISP functions had a similar number of genes (Table 5). There was no clear relation between GC content and gene number per function. In general, genes from the ISP class had lower marginal-GC content than genes from other functional groups (Figure 2) in the core genome of subsp. *fastidiosa* and subsp. *multiplex*, and in the accessory genome of subsp. *pauca*. Genes coding for ribosomal protein were the highest contributors to the lower marginal-GC content in the ISP functional class. Nonetheless, the variable “gene function” still had a significant effect even when these genes were removed from the dataset (Supplementary Table S4). After removal of ribosomal protein-coding genes, marginal-GC content in the CPS and ISP classes was similar in subsp. *fastidiosa* and subsp. *multiplex* core genes (Supplementary Figure S4). Notably, the marginal GC-content of M class genes was lower in the accessory genome, particularly in the case of subsp. *fastidiosa*. Genes from the Poorly characterized (P) category were removed from these visualizations.

**Table 5:** *χ* ^2^ analysis showing statistically significant differences between the number of core and accessory genes from different COG functions within each *Xylella fastidiosa* subspecies

Subspecies	Functional group	Accessory sum	Core sum
*Xylella fastidiosa* subsp. *fastidiosa*	Cellular Processes and Signaling (CPS)	9,214	28,475
	Information Storage and Processing (ISP)	8,159	31,538
	Metabolism (M)	17,018	56,590
	Multiple categories (MU)	1,719	5,301
	Poorly characterized (P)	166,938	124,154
	χ^2^ = 49,336, df = 4, *P* < 2.2 × 10^−16^*		
*Xylella fastidiosa* subsp. *multiplex*	Cellular Processes and Signaling (CPS)	3,086	3,132
	Information Storage and Processing (ISP)	2,906	3,817
	Metabolism (M)	6,186	6,185
	Multiple categories (MU)	572	633
	Poorly characterized (P)	33,890	17,239
	χ^2^ = 2,503.1, df = 4, *P* < 2.2 × 10^−16^*		
*Xylella fastidiosa* subsp. *pauca*	Cellular Processes and Signaling (CPS)	14,599	6,416
	Information Storage and Processing (ISP)	14,937	6,823
	Metabolism (M)	27,773	12,422
	Multiple categories (MU)	2,728	1,043
	Poorly characterized (P)	136,883	25,835
	χ^2^ = 7,591.9, df = 4, *P* < 2.2 × 10^−16^*		

Accessory sum, sum of accessory genes from all isolates within each subspecies; Core sum, sum of core genes from all isolates within each subspecies. Bold values correspond to p-values < 0.05. These values have also been marked with asterisk (*).

* Statistically significant differences.

Gene function had a significant effect on genic GC content even when genes were classified based on their specific function (*i.e.* “Translation,” “Amino acid transport and metabolism,” “Defense mechanism,” etc.) (Supplementary Table S5). The relationship between GC content in core *vs* accessory genes varied across functions, but overall, accessory genes had higher marginal-GC content than core genes of the same function in both subsp. *fastidiosa* and subsp. *multiplex*. This was particularly evident within the ISP functional class, where functions associated with “Translation” and “Replication” had lower marginal-GC content. Differences in nucleotide composition across specific functions were less clear in subsp. *pauca* (Supplementary Figure S5).

**Figure 3:**
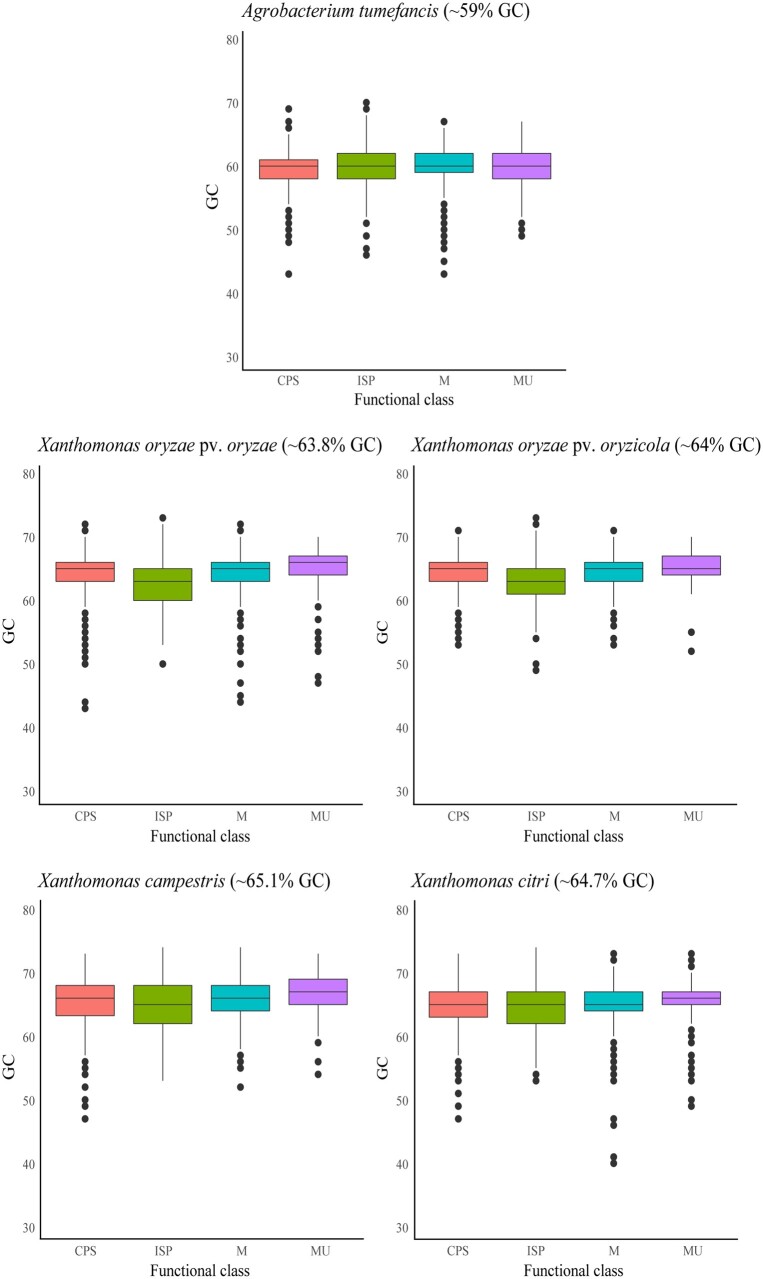
Boxplot showing marginal-GC content distribution on different functional classes in five plant-associated pathogens. Marginal-GC content distribution and average GC content is shown for each phytopathogen: *A. tumefaciens*, *X. oryzae* pv*. oryzae*, *X. oryzae* pv*. oryzicola*, *X. citri*, and *X. campestris*. Functional classes include ISP (green), Cellular Processes and Signaling (CPS, blue), Metabolism (M, red), and Multiple categories (MU, purple).

The distribution of marginal-GC content among functional classes was also evaluated in other plant pathogens (Supplementary Table S6): *A. tumefaciens* (283,876 SNPs and π = 0.059), *X. oryzae* pv*. oryzae* (45,414 SNPs and π = 0.007), *X. oryzae* pv*. oryzicola* (22,029 SNPs and π = 0.002), *X. citri* (111,198 SNPs and π = 0.012), and *X. campestris* (113,771 SNPs and π = 0.011). Neither *X. citri*, *X. campestris*, nor *A. tumefaciens* showed a clear separation in marginal-GC content between functional classes. On the other hand, in both *X. oryzae* pv*. oryzae* and *X. oryzae* pv*. oryzicola* genes from the ISP class had lower marginal-GC content compared with other functional classes, even after removal of ribosomal protein-coding genes (Figure 3). The trend was most prominent in *X. oryzae* pv*. oryzae.* Like in *X. fastidiosa*, functions associated with “Translation” and “Replication” maintained the lowest marginal-GC content.

**Figure 4:**
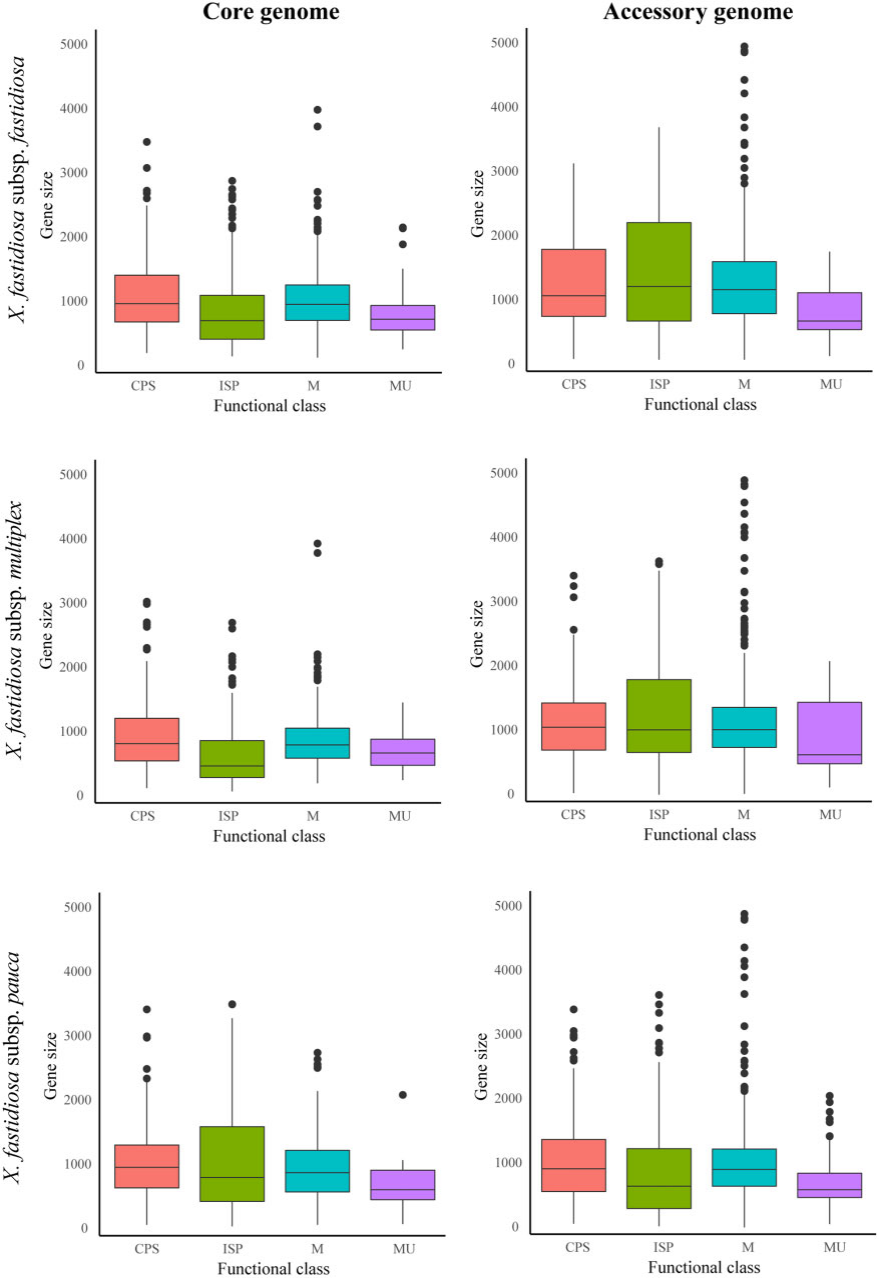
Boxplot showing gene size differences across gene functional classes for three *X. fastidiosa* subspecies. Plots have been divided into core/accessory genes within each *X. fastidiosa* subspecies. Functional classes include ISP (green), Cellular Processes and Signaling (CPS, blue), Metabolism (M, red), and Multiple categories (MU, purple).

**Figure 5:**
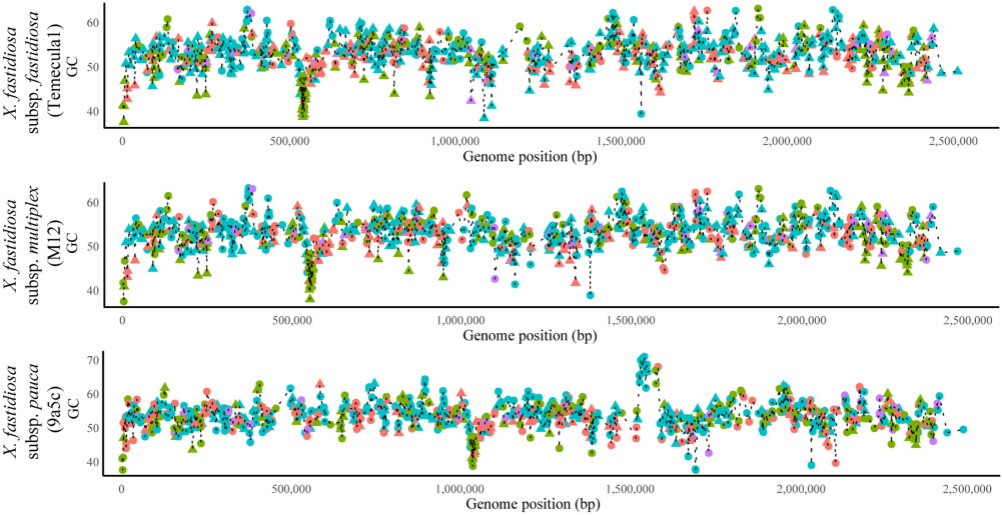
Line plot showing gene-specific GC content *vs* genes position across the length of three finished *X. fastidiosa* assemblies. The assemblies used are subsp. *fastidiosa* (strain Temecula 1), subsp. *multiplex* (strain M12), subsp. *pauca* (strain 9a5c). Core genes are shown with circles while accessory genes are shown with triangles. Functional classes include ISP (green), Cellular Processes and Signaling (CPS, blue), Metabolism (M, red), and Multiple categories (MU, purple).

Genes from the ISP function were smaller than those from other functional groups (Figure 4); however, this difference was largely due to genes coding for ribosomal proteins (Supplementary Figure S6). Similarly, apart from a cluster of ribosomal protein-coding genes, there were no general trends between genic GC content and gene position (Figure 5). A synteny analysis of three complete genome assemblies representing each *X. fastidiosa* subspecies showed three chromosomal inversion events between the 9a5c strain (subsp. *pauca*), and the M12 (subsp. *multiplex*) and Temecula1 (subsp. *fastidiosa*) strains (Supplementary Figure S7).

### Purifying selection is prevalent in genes from the core and accessory genome

Genic GC content and the Tukey’s Ladder of Powers transformation of dN/dS were significantly correlated for individual gene alignments of subsp. *pauca* [*r*(1930) = −0.0558, *P* = 0.0142], but not for subsp. *multiplex* [*r*(3869) = −0.0281, *P* = 0.081] and subsp. *fastidiosa* [*r*(3723) = −0.0247, *P* = 0.1316]. The significant correlation was small. Most individual gene alignments showed signs of gene-wide purifying selection, with only a small proportion having a dN/dS > 1 (subsp. *multiplex* 547/3872, subsp. *fastidiosa* 442/3726, and subsp. *pauca* 226/1933). Most of the genes under positive selection in each subspecies (subsp. *multiplex* 450/556, subsp. *fastidiosa* 393/442, and subsp. *pauca* 162/227) were classified in the P group. In subsp. *fastidiosa*, 12 genes belonged to the CPS class, 15 to the ISP class, and 19 to the M class; in subsp. *multiplex*, 23 genes belonged to the CPS class, 27 to the ISP class, and 50 to the M class; and in subsp. *pauca*, 12 genes belonged to the CPS class, 12 to the ISP class, and 38 to the M class (Supplementary Table S7).

### There are no clear trends in GC content between recombinant and non-recombinant genes

For all analyzed *X. fastidiosa* subspecies, intra-subspecific recombination was pervasive across the length of the core genome alignment and equally frequent among functional groups (Table 6). As a general trend, marginal-GC content was lower in recombinant than non-recombinant genes (Supplementary Figure S8). Core genes within non-recombinant regions had similar lengths, while recombinant genes from the ISP class were slightly smaller in both subsp. *fastidiosa* and *multiplex* (Supplementary Figure S9). Core genes from the Poorly characterized (P) category were removed from the visualizations. Genic GC content and the log10 transformation of r/m were significantly correlated for individual gene alignments of subsp. *multiplex* (*r*(3869) = −0.1516, *P* = 2.2 **×** 10^−16^) and subsp. *fastidiosa* (*r*(3723) = −0.1396, *P* = 2.2 **×** 10^−16^), but not on subsp. *pauca* (*r*(1930) = 0.043, *P* = 0.0588). However, both significant correlations were small.

**Table 6:** χ^2^ analysis showing statistically significant differences between the number of recombinant and non-recombinant genes from different COG functions within each *X. fastidiosa* subspecies

Subspecies	Functional group	Recombinant sum	Non-recombinant sum
*Xylella fastidiosa* subsp. *fastidiosa*	Cellular Processes and Signaling (CPS)	4,721	9,469
	Information Storage and Processing (ISP)	6,253	9,539
	Metabolism (M)	7,299	22,822
	Multiple categories (MU)	507	2,182
	Poorly characterized (P)	21,554	34,491
	χ^2^ = 2277.5, df = 4, *P* < 2.2 × 10^−16^*		
*Xylella fastidiosa* subsp. *multiplex*	Cellular Processes and Signaling (CPS)	188	3,427
	Information Storage and Processing (ISP)	211	4,221
	Metabolism (M)	335	6,972
	Multiple categories (MU)	38	777
	Poorly characterized (P)	625	13,559
	χ^2^ = 4.4525, df = 4, *P* = 0.3482		
*Xylella fastidiosa* subsp. *pauca*	Cellular Processes and Signaling (CPS)	4,721	9,469
	Information Storage and Processing (ISP)	6,253	9,539
	Metabolism (M)	7,299	22,822
	Multiple categories (MU)	507	2,182
	Poorly characterized (P)	21,554	34,491
	χ^2^ = 2277.5, df = 4, *P* < 2.2 × 10^−16^*		

Recombinant sum, sum of recombinant genes from all isolates within each subspecies; Non-recombinant sum, sum of non-recombinant genes from all isolates within each subspecies. Bold values correspond to p-values < 0.05. These values have also been marked with asterisk (*).

* Statistically significant differences.

## Discussion

Compared with its closest relative, *Xanthomonas* spp. (∼5 Mb and 65% GC), *X. fastidiosa* (∼2.5 Mb and ∼51% GC) has undergone significant genomic changes. As in other obligate symbionts (Moran *et al.* 2008; McCutcheon and Moran 2012; Wolf and Koonin 2013), the switch in lifestyle in *X. fastidiosa* was accompanied by a genome reduction. Compositional changes associated with genome reduction are linked to loss of DNA repair genes, which limits the effects of mutational bias toward C/G to A/T transitions (Moran *et al.* 2008; Hershberg and Petrov 2010). This was not the case here, as genes associated with DNA repair functions (*i.e. MutS*, *RadA*, *RecN*, *RecF*, *RecO*, and *AlkA*) were present in *Xanthomonas* spp. and the core/soft-core of *X. fastidiosa*. Repair genes were hypothetically duplicated in the *Xanthomonas-Xylella* ancestor and later lost in *X. fastidiosa* (Martins-Pinheiro *et al.* 2004). This finding, in addition to the higher GC content observed in *Xanthomonas* spp., would suggest that the absence of repair gene duplicates in *X. fastidiosa* facilitated the drop in genome-wide GC content. Previous estimates of the mutation rate of *X. fastidiosa* [7.6 **×** 10^−7^ mutations per site per year; (Vanhove *et al.* 2019)] are larger than those reported for *X. citri* [8.4 **×** 10^−8^ substitutions per site per year; (Richard *et al.* 2020)] and, though not directly comparable, smaller than those reported for *X*. *oryzae* pv. *oryzae* [2 **×** 10^−5^ mutations per gene per year; (Midha *et al.* 2017)]. So, the loss of specific gene repair paralogs in *X. fastidiosa* might have facilitated a species-wide drop in GC content, while the remaining genes aid in maintaining its current nucleotide composition. Yet, not all repair genes are expected to affect GC content (*i.e.* genes involved in base excision repair correct C/G to A/T mutations, while mismatch repair genes do not) (Garcia-Gonzalez *et al.* 2012). Future studies directly assessing the mutation rate in *X. fastidiosa* should be conducted to determine the fidelity of the remaining repair genes.

Another explanation for the drop in GC content observed in *X. fastidiosa* is environmentally imposed nutritional limitations. Shifts to lower GC content are linked to nutrient-limiting environments (Mann and Chen 2010). During energetic constrains, proteins coded using more energetically costly G/C nucleotides are at a selective disadvantage compared with those favoring A/T nucleotides (Rocha and Danchin 2002). This, in addition to an A/T mutational bias, would result in a GC content drop (Mann and Chen 2010). For *X. fastidiosa*, xylem-sap, the primary nutrient source in insect mouthparts and *in planta*, is nutrient limited (Bové and Garnier 2003). In certain xylem-feeding insects, these limitations led to long-term mutualistic associations with *Candidatus Baumannia cicadellinicola* and *Candidatus Sulcia muelleri*, which are housed in bacteriocytes (Braendle *et al.* 2003; Bennett *et al.* 2014); both *C. Baumannia cicadellinicola* (∼20.3% GC) and *C. Sulcia muelleri* (∼22.7% GC) have low GC genomes.

Alternatively, genome reduction is linked to selection-driven gene-loss on accessory genes (Lee and Marx 2012). Recent studies have found that nucleotide composition is more constrained in genes from the core genome compared with the accessory genome (Bohlin *et al.* 2017, 2018). This pattern was also observed here. So, compositional changes in *X. fastidiosa* might be the result of selection-driven gene loss eliminating GC-rich accessory genes. This hypothesis should be tested in studies encompassing a larger range of plant-associated bacteria.

Another point to consider is the unique mutation-selection balance on *X. fastidiosa*. Natural selection can counteract the A/T mutation bias observed in microbial organism (Bohlin *et al.* 2018). In instances where natural selection is limited, genic nucleotide composition would lean toward lower GC values. In *X. fastidiosa*, this could be observed in newly introduced clonal populations. Subsp. *fastidiosa* was introduced to the USA and subsp. *pauca* was introduced to Italy via a single event ∼150 years ago (Vanhove *et al.* 2019)) and ∼17 years ago (Giampetruzzi *et al.* 2017; Vanhove *et al.* 2019), respectively. Yet, genic GC content was only slightly lower in introduced (52.16% GC in Italy and 51.85% GC in the USA) *vs* native populations (52.97% GC in Brazil and 51.91% GC in Costa Rica). Very few studies have assessed transitions and transversion rates in *X. fastidiosa*, with most of them using limited genomic data (Doddapaneni *et al.* 2006). In those using larger datasets, point mutations consistently contribute less to genome diversity than homologous recombination (Scally *et al.* 2005; Rogers and Stenger 2012; Vanhove *et al.* 2019, 2020). While it is unlikely that mutational biases are different in this pathogen compared with others; it is unknown if point mutations in *X. fastidiosa* are limited, if they have been under detected (*i.e.* clonal populations), or if they are masked by other evolutionary forces (*i.e.* recombination). Future studies should leverage the increasing publicly available genome data for this pathogen to explicitly address this issue.

### Nucleotide composition is variable within the accessory genome

GC content was more constrained in the core than the accessory genome. Among accessory genes, the cloud genome (<15% strains) could represent either ancestral gene loss in certain lineages or recent gene gain (Davison 1999; Mira *et al.* 2001). The latter might be more likely since our samples are biased toward newly introduced clonal populations. On the other hand, the soft-core genome (95–99% strains) likely represents recent gene loss events or annotation/assembly errors. Nucleotide composition changes adaptive to a xylem environment (*i.e.* lower GC) would be observed by identifying compositional differences between these groups. Such a trend is observed here.

### Strand-biased nucleotide composition but no strand-biased gene distribution in *X. fastidiosa*

GC content was lower in *X. fastidiosa’*s leading strands. This could be explained by differences in the energetic requirements for *de novo* synthesis of nucleotides and amino acids resulting in strand-biased nucleotide composition (SNC) (Chen and Zhang 2013; Gao *et al.* 2017). In energy-limiting environments and GC-low genomes, SNC favors the lowest energetic cost for protein-coding genes (Wu *et al.* 2012). In most bacteria, mutational bias in the lagging strand favors energetically cheaper nucleotides but more expensive protein products (Rocha 2008; Gao *et al.* 2017). As a result, GC-rich protein-coding genes are preferentially found in the leading strand, particularly if they are highly expressed (Gao *et al.* 2017). Alternatively, replication-transcription conflicts also influence differences in the nucleotide composition of DNA strands. During replication of the lagging strand, RNA and DNA polymerases produce mutations caused by head-on collisions that are more deleterious than the co-directional mutations on the leading strand (Merrikh *et al.* 2012). If AT-biased substitutions are more deleterious in the lagging strand, then the lower GC content in the leading strand of *X. fastidiosa* might be due to the accumulation of A/T mutations otherwise removed from the lagging strand. Nonetheless, the exact mechanism by which replication-transcription conflicts influence genome evolution is still debated. Increase mutagenesis in the lagging strand can be adaptive (Paul *et al.* 2013; Merrikh and Merrikh 2018), or a product of deleterious mutation/purifying selection balance (Chen and Zhang 2013). On the other hand, non-synonymous substitutions are under strong purifying selection in the leading strand (Schroeder *et al.* 2020). Which trend better fits *X. fastidiosa* remains to be determined.

*Xylella fastidiosa* is one of a few bacteria with no significant GC skew (Gregory and DeSalle 2005). However, GC skew analyses have been conducted using few strains, and therefore, subtle differences might not have been detected. Here, we found that the average number of genes in the lagging versus leading strands was 1055 *vs* 1030 for subsp. *fastidiosa*, 1096 *vs* 1095 for subsp. *pauca*, and 1127 *vs* 1079 for subsp. *multiplex*. Contrary to our observation, estimations based on the predicted location of the origin and terminus of replication projected that 59–60% of *X. fastidiosa* genes would be located on the leading strand (Retchless *et al.* 2014). These estimates were obtained using five finished and three draft quality *X. fastidiosa* genome sequences. Therefore, trends specific to *X. fastidiosa* might have not been observed. Also, nucleotide compositional changes driven by pressures for rapid and efficient gene expression might not be predominant in slow-growing bacteria such as *X. fastidiosa* (Bustamante Smolka *et al.* 2003). This would result in a similar number of genes in either DNA strand as seen here. Bacterial chromosomes lacking the DNA polymerase III alpha subunit *polC* (such as *X. fastidiosa*) also have less significant strand-biased gene distribution (Wu *et al.* 2012). Finally, G/T and A/T nucleotide combinations were predominant in the leading strand, while A/C and G/C combinations were predominant in the lagging strand. This suggests that, in *X. fastidiosa*, T bases have strong dominance in the leading strand, followed by G bases. This matches other non-*polC* genomes (Gao *et al.* 2017) as well as predictions based on mutational and selective pressures (Zhang and Gao 2017).

### Gene function, size, and location have an interlinked effect on *X. fastidiosa* GC content

Genes from the ISP functional class, particularly in the subsp. *fastidiosa* and *multiplex* core genomes, were shorter and had lower GC content compared with other genes. The differences were largely the result of a clustered group of ribosomal protein-coding genes. After their removal, both CPS and ISP genes had similar GC content distribution. In *X. fastidiosa*, survival is tightly linked to efficient replication and movement within xylem vessels (Sicard *et al.* 2018). Genes involved in “Translation” and “Replication” often had lower GC content than other bacterial genes. Codons that facilitate mRNA folding into a more unstable secondary structure are thought to enable efficient translation initiation (Gu *et al.* 2010; Bentele *et al.* 2013). Also, repetitive AT segments and genes with lower GC content found near the replication origin (*ori*C), facilitate the opening of the DNA double helix and the initiation of replication (Rajewska *et al.* 2012; Li *et al.* 2014). Functions associated with “Cell cycle control” and “Signal transduction,” both involved in sensing and responding to external signals (Skerker *et al.* 2005), also had lower GC content. Our results suggest that nucleotide compositions facilitating replication and growth are favored.

The clustered organization of ribosomal protein genes (ribosomal superoperons) matches that previously described in other bacteria. Such an organization facilitates control during transcription and translation (Lecompte *et al.* 2002; Fox *et al.* 2009; Yutin *et al.* 2012). In fast-growing bacteria, ribosomal protein genes are located near the *ori*C, as a mechanism to secure more ribosomal proteins, facilitate ribosome assembly, and enable translation (Soler-Bistué *et al.* 2015). Though ribosomal proteins were clustered in *X. fastidiosa*, they were not closely associated with the estimated start of the replication in complete genome assemblies fitting expectations for slow-growing bacteria. The ribosomal supercluster was closer to the *ori*C in subsp. *fastidiosa* (∼536,660–549,051) and subsp. *multiplex* (∼547,934–561,894 bp), compared with subsp. *pauca* (∼1,109,361–1,121,748 bp). Whether this indicates adaptive changes facilitating rapid replication in some *X. fastidiosa* subspecies or is the result of architectural chromosome changes (though the cluster is outside three major inversion events) remains to be evaluated.

Outside *X. fastidiosa*, ISP-linked differences in GC content were only found in *X. oryzae* pv*. oryzae* and *X. oryzae* pv*. oryzicola.* Moreover, ISP genes had lower marginal-GC content in *X. oryzae* pv*. oryzae* than in *X. oryzae* pv*. oryzicola.* The mechanisms to invade plant tissues are different in each pathovar (*i.e.* vascular tissue *vs* plant parenchyma) (Niño-Liu *et al.* 2006)*.* Gene gain/loss events have mediated the transition between vascular and non-vascular pathovars (Gluck-Thaler *et al.* 2020). Therefore, other genomic variables (*i.e.* nucleotide composition, genome architecture, or gene duplication) could also be associated with the mechanisms of plant infection. Xylem-limited pathogens move through long distances in a nutrient-limited environment and cause systemic infection while non-vascular pathogens remain restricted to infection sites formed by living parenchyma cells (Mensi *et al.* 2014). In this regard, the nutritional limitations imposed by the host environment would be different between pathovars, which could lead to variations in genomic GC content. Another point to consider is that different ISP genes might have distinct evolutionary origins due to genome rearrangement and duplication events. Further analyses focused on *Xanthomonas* spp. should be conducted to address this question.

### Natural selection was associated with gene-specific GC content only in subsp. *pauca*

Purifying selection was predominant in *X. fastidiosa* and dN/dS values were similar regardless of the number of ortholog sequences within gene alignments. Previous reports (Bohlin *et al.* 2017) have found that purifying selection limits the amount of viable variation in genes that are essential for survival, particularly within the core genome. In accordance, our results show that most non-synonymous changes are deleterious. *Xylella fastidiosa* is thought to have undergone genome reduction (Simpson 2000; Rodriguez-R *et al.* 2012) and developed a complete, but minimalist, metabolic network (Kurokawa *et al.* 2016; Gerlin *et al.* 2020). This and its recent association with multiple crops (Rapicavoli *et al.* 2018; Sicard *et al.* 2018) could have resulted in limitations to *X. fastidiosa* genetic variation. Only few groups of ortholog genes showed signs of positive selection (dN/dS > 1). Most of them belonged to the M class. A less conservative analysis (*e.g.* a branch-site selection test) might highlight a different pattern, yet, these results indicate that metabolic adaptation is occurring in *X. fastidiosa.*

Genes favoring higher dN/dS had lower GC content in subsp. *pauca*. However, we could not establish if GC content was differently favored between non-synonymous *vs* synonymous changes from our results. Within bacteria, natural selection favors increased synonymous GC content (Hildebrand *et al.* 2010; Raghavan *et al.* 2012); so, we expect that a similar trend would be observed in *X. fastidiosa*. The negative relation between GC content and dN/dS values observed in subsp. *pauca* could suggest a decrease in GC content favored following a drop associated, perhaps, with its genome reduction. Yet, this correlation (*r*(1930) = −0.0558, *P* = 0.0142) was small and future studies should determine its biological relevance, as well as address the potential role of gene expression and transcription in GC content. In addition, it should be noted that in the case of subsp. *pauca* and subsp. *fastidiosa*; a significant proportion of our data originates from recently introduced populations. This could limit the action of natural selection due to a recent founder effect.

### Intra-subspecific recombination has a variable effect in *X. fastidiosa* GC content

Recombination occurs between sympatric subsp. *pauca* strains (Almeida *et al.* 2008; Coletta-Filho *et al.* 2017; Francisco *et al.* 2017), has a phylogenetic and geographic component (Nunney *et al.* 2013, 2014a, 2014b; Kandel *et al.* 2017; Landa *et al.* 2020), and plays a role in speciation (Nunney *et al.* 2014a) and host switching (Nunney *et al.* 2014b; Coletta-Filho *et al.* 2017; Vanhove *et al.* 2019). Yet, our results indicate that intra-subspecific recombination does not have a distinct effect on nucleotide composition. Recombinant core genes had lower GC content than non-recombinant core genes, though this was not observed when genes were subdivided according to function. This suggests that the differences in nucleotide composition among functional groups are not caused by recombination, at least in the core genome. In addition, while *r*/*m* rates were significantly correlated with GC content in subsp. *multiplex* (*r*(3869) = −0.1516, *P* = 2.2 **×** 10^−16^) and subsp. *fastidiosa* (*r*(3723) = −0.1396, *P* = 2.2 **×** 10^−16^) the relationships were small. Whether these trends are biologically meaningful or not, they indicate that homologous recombination might play a role in mediating the nucleotide composition of the accessory genome.

Previous studies have found that GC-biased gene conversion (gBGC) results in increased GC content in recombinant genes (Lassalle *et al.* 2015). Alternatively, GC content decreases within recombinant regions of highly recombinant genomes (Suerbaum *et al.* 1998; Falush *et al.* 2001; Lassalle *et al.* 2015). A study evaluating 54 bacterial genomes with diverse lifestyles (*i.e.* endosymbionts and intracellular pathogens, opportunistic pathogens, commensal and free-living bacteria, and obligate pathogens), found that nucleotide composition did not differ between recombinant and non-recombinant regions (González-Torres *et al.* 2019). While this study focused largely on bacterial species of medical interest, the three plant pathogens included (*X. campestris*, *X. oryzae*, and *X. fastidiosa*) did not show any differentiating trends. The analysis conducted here confirms these results in *X. fastidiosa’*s core genome but does not eliminate the possibility nucleotide composition in the accessory genome might be linked to recombination.

## Conclusion

*Xylella fastidiosa* has undergone significant biological, ecological, and genomic changes. Thus, it can be a valuable organism to better understand nucleotide compositional changes in bacterial plant symbionts. Our results indicate that GC content has dropped in *X. fastidiosa* compared with its closest relative. Several hypotheses are presented to explain this drop and should be tested further. Yet, particular focus should be dedicated to better understanding the mutation rate of *X. fastidiosa.* For this pathogen species, changes in nucleotide composition do exist but are small. It is notable that nucleotide composition of most of the genome of *X. fastidiosa* is conserved regardless of numerous variables having a statistical effect on it. We hypothesize that distinct evolutionary forces and energetic constraints both drive and limit these small variations. For example, recombination in the core genome and purifying selection would limit the number of nucleotide changes; while recombination in the accessory genome, nutritional limitations in the environment, and mutation/selection biases drive genic GC content drops. Taken together, we show that even in a GC-balanced genome like that of *X. fastidiosa*, nucleotide changes are observed, and the action of evolutionary forces can be detected.
